# Randomized Controlled Trial of Laparoscopic versus Open Radical Cystectomy in a Laparoscopic Naïve Center

**DOI:** 10.1155/2021/4731013

**Published:** 2021-07-07

**Authors:** Waleed Mohamed Fadlalla, Ayman Hanafy, Mahmoud Abdelhakim, Hatem Aboulkassem, El Sayed Ashraf, Ahmed Abdelbary

**Affiliations:** ^1^National Cancer Institute, Cairo University, Department of Surgical Oncology, Cairo, Egypt; ^2^Department of Urology, Faculty of Medicine, Cairo University, Cairo, Egypt

## Abstract

**Background:**

Laparoscopic radical cystectomy is a challenging surgical procedure; however, it has been largely abandoned in favor of the more intuitive robotic-assisted cystectomy. Due to the prohibitive cost of robotic surgery, the adoption of laparoscopic cystectomy is of relevance in low-resource institutes. *Methodology*. This is a randomized controlled trial comparing laparoscopic radical cystectomy (LRC) to open radical cystectomy (ORC) at a single institute. Each group included thirty patients. The trial was designed to compare both approaches regarding operative time, blood loss, transfusion requirements, length of hospital stay, time to oral intake, requirement of opioid analgesia, and complications.

**Results:**

LRC was associated with less hospital stay (9.8 vs. 13.8 days, *P*=0.001), less time to oral solid intake (6 vs. 8.6 days, *P*=0.031), and lower opioid requirements (23.3% vs. 53.3%, *P*=0.033). There was a trend towards lower blood loss and transfusion requirements, but this did not reach statistical significance. Overall complication rates were comparable.

**Conclusion:**

Laparoscopic radical cystectomy was associated with comparable postoperative outcomes when compared to ORC in the first laparoscopic cystectomy experience in our center. Benefitting from the assistance of an experienced laparoscopic surgeon is recommended to shorten the learning curve.

## 1. Introduction

In Egypt, bladder cancer is the third most common solid malignancy and the second most common cancer in males after liver cancer [1], representing a major health problem. The standard for the surgical management of muscle invasive bladder cancer (MIBC) and selected cases of nonmuscle invasive bladder cancer (NMIBC) is open radical cystectomy. Radical cystectomy is associated with 30–68% complication rates in contemporary series [2, 3]. Few procedures in urology (if any) are associated with the morbidity of radical cystectomy; improvement of results in that domain represents a priority in urologic literature.

The first laparoscopic radical cystectomy was performed in 1992 [4]. The adoption of laparoscopic radical cystectomy was slow due to the challenging nature of the procedure. In addition, the introduction of robotic surgery as a minimally invasive interface for radical cystectomy was a more appealing option, as the robot was designed to remedy the difficulties of conventional laparoscopy and shorten the learning curve required to master the procedure [5]. Laparoscopic cystectomy is performed within a confined space with little room for surgical manipulation; this adds to the difficulty when attempting bladder surgery which is surrounded by a rich vascular network, as well as dissection around great vessels during lymphadenectomy, with the potential for life-threatening injury.

While the robot is an extremely valuable tool, alternatives should be sought after, especially in a low-resource setting. Ideally, these alternatives should not compromise the quality of care provided to the patients. In this study, we explore the feasibility, safety, and postoperative outcome of laparoscopic radical cystectomy in a laparoscopic-naïve center.

## 2. Methodology

### 2.1. Study Design

This is a randomized, controlled, unblinded interventional clinical trial comparing open radical cystectomy to laparoscopic radical cystectomy. Operations in the intervention arm were performed by the same surgical team with one of three experienced open surgeons as the operator and the others assisting. One of two expert oncosurgeons performed all the procedures in the control arm (open radical cystectomy). A parallel assignment intervention model with 1 : 1 allocation was used in this study. The trial was registered with ClinicalTrials.gov (NCT04838873).

### 2.2. Pilot Study

A pilot of 10 patients was performed utilizing the presence of a urologist having an immense experience with laparoscopic radical cystectomy. This helped organize the team, adjust the setup, troubleshoot early problems, and build up confidence.

### 2.3. Participants

**Figure 1:**
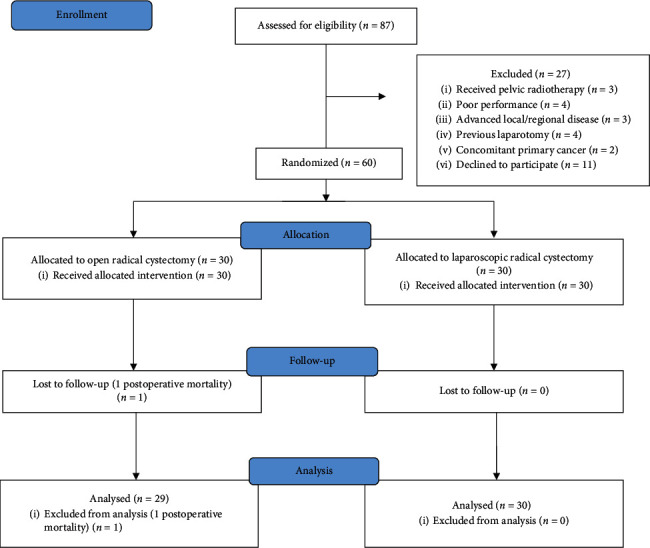
Flowchart of the study.

Patients scheduled for radical cystectomy at the National Cancer Institute, Cairo University, between January 25th, 2019, and July 30th, 2019, were recruited and randomized to one of two study groups (a control group and an intervention group) (Figure 1). Both groups were matched in terms of preoperative parameters, including preoperative serum creatinine levels, hemoglobin values, and clinical T and N stages.

### 2.4. Selection Criteria

#### 2.4.1. Inclusion Criteria

- Patients diagnosed with urinary bladder cancer, clinical stage T3a, N0, M0 or less [6], scheduled for radical cystectomy
- 30–80 years of age
- Able to comprehend and sign informed consent

#### 2.4.2. Exclusion Criteria

The following exclusion criteria were predefined:

- Patients with nodal and/or distant metastasis
- Patients with severe medical comorbidities contraindicated for major surgery
- Patients with uncorrectable bleeding diathesis
- Patients with previous extensive abdominal surgery/adhesions precluding laparoscopic access
- Patients who received preoperative radiation therapy to the pelvis

### 2.5. Intervention

#### 2.5.1. Surgical Technique (Control Group)

A standard lower midline laparotomy was used for either conventional open radical cystoprostatectomy in male patients or anterior pelvic exenteration in females.

#### 2.5.2. Surgical Technique (Study Group)

**Figure 2:**
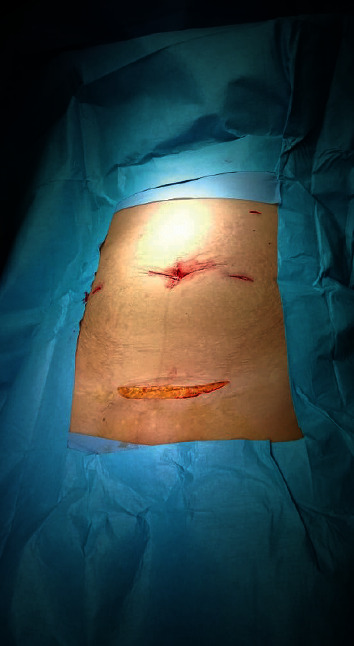
Pfannenstiel incision is used to deliver the specimen and perform urinary diversion.

Transperitoneal approach to laparoscopic radical cystectomy was performed as described in literature [7]. A muscle-splitting Pfannenstiel incision was utilized to deliver the specimen and perform the diversion (Figure 2).

### 2.6. Data Collection and Outcome Measures

Baseline characteristics, operative variables, complications, and pathologic outcomes will be discussed in detail in the “Results” section. All clinical and pathological information was recorded prospectively, with patient datasheets filled out by healthcare personnel. Both groups were matched in terms of preoperative parameters, including preoperative serum creatinine levels, preoperative hemoglobin values, body mass index (BMI), and clinical T and N stages (Table 1).

### 2.7. Randomization and Blinding

Randomization was generated using the SPSS. Both the patient and the operating surgeon knew the allocated intervention before the surgery. However, the study statistician performing the final analysis was masked to the interventions.

### 2.8. Statistical Analysis

#### 2.8.1. Sample Size Estimation

This study aims to compare laparoscopic radical cystectomy with open radical cystectomy regarding perioperative outcome. Based on a previous study by Lin and colleagues [8], the mean operative time for LRC was 282 minutes for LRC vs. 235 for ORC. Comparable results were observed in our pilot of 10 patients. Thus, a total sample of 26 patients per group will be sufficient to detect a power of 90%, significance level of 0.05. The number was increased to a total of 30 patients per group to allow for the use of parametric tests and account for possible drop-outs. Sample size estimation was performed by G power sample size program (*Franz Faul, University of Kiel, Germany*).

#### 2.8.2. Statistical Methods

Data were analyzed using SPSS statistical package version 22 (*SPSS Inc., Chicago, IL*). Numerical data were expressed as mean and standard deviation (SD), median, and range as appropriate. Qualitative data were expressed as frequency and percentage. Student *T*-test was used to examine the relation between quantitative variables as appropriate. Chi-square (Fischer's exact) test was used to examine the relation between qualitative variables as appropriate. Tests were two-tailed and *P* values ≤0.05 were considered statistically significant.

### 2.9. Ethical Standards

The study adhered to the World Medical Association Declaration of Helsinki and the ethical standards of the National Cancer Institute, Cairo University. IRB full approval was obtained prior to the initiation of the study (IRB number: 201819018.3) (Study ID: S01901-31004), as well as written informed consent from all participants.

### 2.10. Follow-Up

Patients were followed for at least 1 year postoperatively. Regular follow-up visits were scheduled at 1 week after discharge, the third month, the sixth month, and the twelfth month unless the patient required closer follow-up for specific complaints.

## 3. Results

### 3.1. Baseline Characteristics

At the first postoperative day, no statistically significant change in serum creatinine or hemoglobin levels was detected (Table 1).

### 3.2. Perioperative Parameters and Complications

#### 3.2.1. Conversion to Open Surgery

No conversions to open surgery were encountered. However, in the prior pilot study, we encountered 3 conversions (3/10 cases, 30%), one due to extensive intra-abdominal adhesions and the other two due to grossly enlarged iliac lymph nodes.

#### 3.2.2. Blood Loss and Transfusion

There was a trend towards decreased blood loss and transfusion requirements in favor of the laparoscopic approach; however, it did not reach statistical significance **(**Table 2**)**.

#### 3.2.3. Operative Time

**Figure 3:**
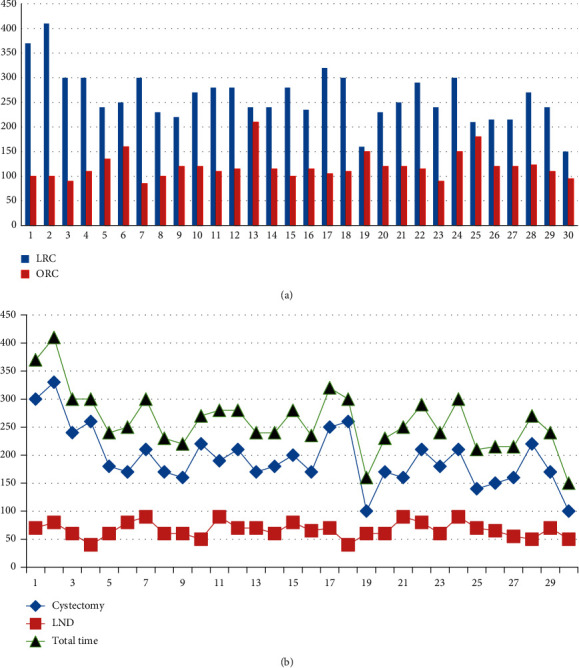
(a) Comparison of operative duration between open and laparoscopic cystectomy. (b) The difference in OR time between the first 15 laparoscopic cases (mean: 280 minutes ± 52) and the last 15 laparoscopic cases (mean: 242 minutes ± 49) (*P*=0.0488).

In our pilot of 10 patients undergoing laparoscopic radical cystectomy, average operative time was 389 minutes (range: 215–455). In the study itself; difference in operative (OR) time (cystectomy and lymphadenectomy time) was statistically significant, with longer operative times in the laparoscopic cystectomy group (Table 3, Figure 3(a)). The difference in OR time between the first 15 laparoscopic cases and the last 15 cases was statistically significant (*P*=0.0488) (Figure 3(b)).

#### 3.2.4. Types of Urinary Diversion

There was no difference regarding the trend of urinary reconstruction in either group (Table 1).

#### 3.2.5. Narcotic Requirements, Time to Solid Intake, and Length of Hospital Stay (LOS)

Regarding laparoscopic radical cystectomy, time to oral solid food intake and length of hospital stay were shorter when compared with open radical cystectomy. Postoperative opioid requirement was also significantly lower for laparoscopic radical cystectomy (Table 4).

#### 3.2.6. Complications

Ileus was more pronounced with ORC (*P*=0.02). There was no difference regarding any type of complications between both study groups. There was one mortality in the ORC group, from pulmonary embolism secondary to left lower limb deep vein thrombosis. More hospital readmissions were observed in the ORC group, but this was not statistically significant (Table 4).

### 3.3. Pathologic Outcome

Only one patient developed a positive surgical margin from the ORC group; no patients from the LRC developed positive margins. No statistically significant differences regarding pathologic T stage or lymph node involvement were reported. Lymph node yield was higher in the LRC group. Twelve patients developed recurrence/metastasis during the follow-up period (Table 5).

## 4. Discussion

Our institute has been one of the main hospitals managing bladder cancer in the country since the seventies; however, we have not adopted laparoscopic surgery until recently, and it was primarily reserved for adrenalectomies and nephrectomy for small renal masses.

### 4.1. Operative Time

This study showed that the operative time was significantly longer in the LRC group compared to the ORC group (366 vs. 229 min; *P* < 0.001), being consistent with literature, which almost uniformly reports operative time to be longer with minimally invasive approaches [6, 9, 10]. This is our early experience with laparoscopic radical cystectomy, and even though the operative duration difference was significant between the open and laparoscopic approaches, there was a trend towards a reduction of operative time when comparing the first and the last laparoscopic cases (370 minutes for the first case vs. 150 minutes for the last case); that difference was statistically significant and reflects the improvement in operative efficiency as challenges within the learning curve are gradually overcome.

### 4.2. Conversion to Laparotomy

It is reported that the rate of open conversion of laparoscopic radical cystectomy ranges from 2 to 6% [11–13]. No cases were converted to open surgery in our study; however, three cases were converted in the prior pilot study (3/10 cases, 10%), one due to extensive intra-abdominal adhesions causing lack of progress and the other two due to the presence of grossly enlarged iliac lymph nodes. The pilot study might have helped add to our vigilance in obtaining recent abdominal-pelvic scans before operation. A low threshold for continuing laparoscopy in advanced bladder cancer should be exercised due to concerns about compromising oncologic control as a result of potential pneumoperitoneum-induced circulation of tumor cells. An expert laparoscopic surgeon was present as a mentor in all cases and scrubbed in if required, for any encountered technical difficulty; this might have reduced the conversion rate further.

### 4.3. Blood Loss and Transfusion Requirements

There was an overall trend towards lower estimated blood loss and lower transfusion rate in LRC group, which is consistent with other studies [7, 8, 14], but this was statistically nonsignificant (*P*=0.119 and *P*=0.207, respectively). Additionally the rate of transfusion of more than 1 unit of RBCs was higher in the open cystectomy group (11 cases vs. 4 cases). The lack of a statistically significant difference might be attributed to the fact that the cystectomy cases in the control group were performed by two highly open experienced surgeons well beyond their learning curves. If the laparoscopic interface allows surgeons in their early laparoscopic experience to achieve similar or even slightly more favorable blood loss rates and transfusion requirements to experienced open surgeons, this could be considered an advantage for minimally invasive surgery. Laparoscopy adds certain benefits allowing for lower blood loss: the field is magnified which allows preemptive control of small potential bleeders especially in areas of restricted exposure, pneumoperitoneal pressure allows for control of small venous oozing, and lastly the surgeon is compelled to operate in a bloodless environment to enhance their visualization.

### 4.4. Opioid Requirements

Lower narcotic requirement is consistently reported with minimally invasive radical cystectomy when compared to open surgery [5, 10]. Our study reports similar results; even though we used a muscle-splitting Pfannenstiel incision for specimen delivery and extracorporeal diversion, the duration of abdominal wall retraction is much less than in a completely open radical cystectomy. Studies suggest that midline laparotomy is more painful than transversely oriented incisions [15], which might be another explanation of the reduced opioid requirements in the LRC group.

### 4.5. Start of Oral Solids and Length of Hospital Stay

Time to regular oral diet and length of hospital stay were significantly shorter for LRC compared with the ORC group, which was consistent with many reports [7, 8, 16]. Open radical cystectomy involves packing of the intestine which potentially exposes them to mechanical trauma for the whole duration of the procedure [17]; this may translate into ileus which is reported in 26% of patients in literature [16].

### 4.6. Complications

According to Challacombe et al., major complication rates ranged from 10 to 13% in LRC [18]. In our trial, we did not find a high rate of complications specific to the laparoscopic approach. This may be because urinary diversions were constructed extracorporeally in all cases, which is a safe and effective way to decrease operative time and surgical complexity. Mortality after radical cystectomy is reported in under 5% [19]. One case died in our study in the ORC group due to pulmonary embolism complicating DVT, with no significant difference between both study groups.

Wound complications including infection, seroma, and dehiscence were not statistically significant between both groups. Important potential advantages of transverse incisions are that they are cosmetically favorable, less liable to evisceration, and associated with less pain and pulmonary compromise [11, 20–22]. Although this is not a consistent finding in literature [23, 24], in this study the only advantage of the transverse incision was the lower opioid requirement in favor of laparoscopic cystectomy.

Hospital readmission was required in seven patients (11.7%; 2 from the LRC and 5 from the ORC groups, respectively, *P*=0.42). This was in line with other studies which reported a relatively high readmission rate of 8.6–28% after radical cystectomy [25, 26]. In a large cohort of 8827 Medicare patients, there was no difference in rates of hospital readmissions between minimally invasive and open radical cystectomy, which suggests that the high readmission rates are inherent to radical cystectomy itself regardless of the approach [27].

### 4.7. Influence of the Approach on the Choice of Diversion

There were no differences in the trend of urinary tract reconstruction between both groups. A well-performed laparoscopic radical cystectomy can replicate open surgery principles and allow for nerve preservation and urethral complex sparing as prerequisites for orthotopic reconstruction. The Pfannenstiel incision does not hinder the creation of any type of neobladder (performed in 4 cases). While the number of neobladder reconstructions was few in both groups, laparoscopic radical cystectomy did not seem to influence the choice of diversion.

### 4.8. Pathologic Metrics of Adequate Resection

#### 4.8.1. Surgical Margins

According to Chade et al., the incidence of positive surgical margins ranged from 4 to 5% of ORC cases and from 0 to 5% of the LRC cases [28]. Only one patient from the control arm developed a positive surgical margin in our study with no statistically significant difference. These findings are similar to those of previous studies [9, 29].

#### 4.8.2. Lymph Node Count

LRC showed a statistically significant higher mean lymph node yield 15.67 (LN) than ORC 11.97, which was contradictory to other relevant reports [5, 7]. Most studies reported similar or higher nodal yields with ORC. The CORAL trial showed that lymph node yield was higher with ORC when compared to LRC (18.8 vs. 15.5) [16]. We did not attempt extended lymph node dissection in any case in the ORC group. However, we could not find an explanation for the higher lymph node yield reported for LRC in our study, other than the fact that the laparoscopic arm was performed by experienced open surgeons. Nevertheless, lymph node yield is considered a measure of adequate staging and regional control in radical cystectomy, and the laparoscopic approach did not compromise lymph node yield in our study.

## 5. Strengths and Limitations

Limitations of our study include short follow-up with lack of oncologic evaluation and the relatively small single institution sample. Both the patients and the surgeon could not be blinded because of the surgical nature of the trial. Strengths include randomized design, all patients adhered to their follow-up schedule with no drop-outs, the open radical cystectomy arm was used by two expert oncosurgeons, and the laparoscopic arm was utilized by the same team throughout the study. This study is also of relevance to other institutes with limited laparoscopic experience aiming to start a laparoscopic cystectomy program.

## 6. Conclusion

Our results show that laparoscopic radical cystectomy is associated with satisfactory short-term outcome, even in an institute with limited laparoscopic experience. Lower narcotic requirements, less time to oral intake and earlier convalescence, were observed in the current study. Longer follow-up is required to monitor oncologic results. We recommend benefiting from the assistance of an experienced laparoscopic surgeon in early series of advanced laparoscopic surgery.

## Figures and Tables

**Table 1:** Baseline patient characteristics.

		Whole cohort N = 60	LRC N = 30	ORC N = 30	*P* value
Age (years)		59.02 ± 7.55	57.20 ± 8.38	60.83 ± 6.24	0.062
Follow-up (months)		20.5 (16–24)	20.4 (16–24)	20.7 (17–24)	0.6
Sex	Male	47 (78.3%)	22 (73.3%)	25 (83.3%)	0.532
	Female	13 (21.7%)	8 (26.7%)	5 (16.7)	
ASA	1	22 (36.7%)	12 (40%)	10 (33.3%)	0.838
	2	35 (58.3%)	17 (56.7%)	18 (60%)	
	3	3 (5%)	1 (3.3%)	2 (6.7%)	
Body mass index (BMI)		24.9 ± 2 (range: 20–28.5)	24.9 ± 1.8 (range: 20.5–28)	24.8 ± 2.1 (range: 20–28.5)	0.8
Previous abdominal surgery		8 (13.3%)	4 (13.3%)	4 (13.3%)	1.00
Neoadjuvant chemotherapy		22 (36.7%)	14 (46.7%)	8 (26.7%)	0.18
SCr preoperative (mg/dl)		1.12 ± 0.30	1.08 ± 0.25	1.15 ± 0.34	0.323
SCr 1st postoperative day (mg/dl)		1.08 ± 0.19	1.03 ± 0.15	1.14 ± 0.20	0.026
Hb preoperative (gm/dl)		12.00 ± 1.50	12.01 ± 1.46	11.98 ± 1.57	0.932
Hb 1st postoperative day (gm/dl)		10.62 ± 1.25	10.70 ± 1.24	10.53 ± 1.29	0.611
Clinical T stage	cT1	10 (16.7%)	5 (16.7%)	5 (16.7%)	0.920
	cT2	34 (56.7%)	18 (60%)	16 (53.3%)	
	cT3	16 (26.7%)	7 (23.3%)	9 (30%)	
Clinical N stage	cN0	49 (81.7%)	23 (76.7%)	26 (86.7%)	0.488
	cN1-3	11 (18.3%)	7 (23.3%)	4 (13.3%)	
Diversion	Orthotopic	9 (15%)	4 (13.3%)	5 (16.7%)	0.99
	Ileal conduit	51 (85%)	26 (86.7%)	25 (83.3%)	

LRC: laparoscopic radical cystectomy; ORC: open radical cystectomy; ASA: American Society of Anesthesiologists' classification; SCr: serum creatinine; Hb: hemoglobin. *P* value ≤0.05 is considered significant. Values are given in means ± SD or *n* (%).

**Table 2:** Comparison of intraoperative estimated blood loss and required blood transfusion units between the LRC and ORC groups.

		LRC *N* = 30	ORC *N* = 30	*P* value
Estimated blood loss (ml)		437.33 ± 374.24	602.67 ± 432.44	0.119
RBCS transfusion (unit)	0	23 (76.7%)	16 (53.3%)	0.207
	1	3 (10%)	3 (10%)	
	2	2 (6.7%)	8 (26.7%)	
	3	1 (3.3%)	2 (6.7%)	
	4	1 (3.3%)	1 (3.3%)	

**Table 3:** Comparison of cystectomy and lymphadenectomy operative time (minutes) between the LRC and ORC groups.

	Whole cohort *N* = 60	LRC *N* = 30	ORC *N* = 30	*P* value
Total operative time (min)	311.7 ± 110.5	394.8 ± 94.8	228.6 ± 39.2	<0.001
Cystectomy operative time (min)	129.6 ± 76.2	194.7 ± 51.2	64.5 ± 20.9	<0.001
Lymphadenectomy operative time	61.1 ± 15	66.5 ± 14	55.7 ± 14.2	0.002

**Table 4:** Comparison of postoperative outcomes and complications between LRC and ORC groups.

	Whole cohort (*N* = 60)	LRC (*N* = 30)	ORC (*N* = 30)	*P* value
Time to solids oral intake (days)	7.35 ± 4.64	6.07 ± 3.62	8.63 ± 5.22	0.031
LOS (days)	11.82 ± 4.83	9.8 ± 4.13	13.83 ± 4.69	0.001
Postoperative opioid requirement	23 (38.3%)	7 (23.3%)	16 (53.3%)	0.033
Conversion from laparoscopy to open	0	0	N/A	
Seroma	7 (11.7%)	2 (6.7%)	5 (16.7%)	0.4
Surgical site infection (SSI)	3 (5%)	1 (3.3%)	2 (6.7%)	0.99
Urinary tract infection	1 (1.7%)	1 (3.3%)	0	0.99
Ileus	10 (16.7%)	2 (6.7%)	8 (26.7%)	0.02
Urine leak	4 (6.7%)	2 (6.7%)	2 (6.7%)	0.99
Small intestinal injury	0	0	0	0.99
Rectal injury	1 (1.7%)	0	1 (3.3%)	0.99
Anastomotic bowel leak	4 (6.7%)	2 (6.7%)	2 (6.7%)	0.47
Abdominal wall dehiscence	3 (5%)	0	3 (10.3%)^*∗*^	0.24
Pneumonia	2 (3.3%)	0	2 (6.7%)	0.47
Pulmonary embolism	2 (3.3%)	1 (3.3%)	1 (3.3%)	0.99
Hospital readmission	7 (11.7%)	2 (6.7%) (i) Small intestinal leakage (ii) DVT	5 (17.2%)^*∗*^ (i) Small intestinal leakage (2 cases) (ii) Abdominal wall dehiscence (2 cases) (iii) Small bowel obstruction (1 case)	0.4
Mortality	1 (1.7%)	0	1 (3.3%)	0.99
Incisional hernia	3	0	3/29 (10.3%)	0.2
Port-site recurrence	0	0	N/A	

DVT: deep vein thrombosis; LOS: length of stay; LRC: laparoscopic radical cystectomy; ORC: open radical cystectomy; N/A: not applicable; ^*∗*^Total number of cases is 29 due to the presence of one postoperative mortality in the ORC group.

**Table 5:** Pathologic outcome.

		Whole cohort *N* = 60	LRC *N* = 30	ORC *N* = 30	*P* value
Pathological T stage	pT0	5 (8.3%)	5 (16.7%)	0	0.098
	pT1	3 (5%)	1 (3.3%)	2 (6.7%)	
	pT2a	4 (6.7%)	1	3 (10%)	
	pT2b	6 (10%)	2 (6.7%)	4 (13.3%)	
	pT3a	10 (16.7%)	7 (23.3%)	3 (10%)	
	pT3b	25 (41.7%)	11 (36.7%)	14 (46.7%)	
	pT4a	7 (11.7)	3 (10%)	4 (13.3%)	
Positive margin		1 (1.7%)	0	1 (3.3%)	0.99
LNs no.		13.82	15.23 ± 4.14	11.97 ± 5.48	0.008
Recurrence		12 (20.3%)^*∗*^	5 (16.7%)	7 (24.1%)^*∗∗*^	0.7

^*∗*^Total number of cases is 59 due to the presence of one postoperative mortality in the ORC group. ^*∗∗*^Total number of cases is 29 due to the presence of one postoperative mortality in the ORC group.

## Data Availability

Data are available upon request which can be directed to the corresponding author.
